# Correlation of Peripheral Blood Parameters and Immune-Related Adverse Events with the Efficacy of Immune Checkpoint Inhibitors

**DOI:** 10.1155/2021/9935076

**Published:** 2021-05-10

**Authors:** Rilan Bai, Lingyu Li, Xiao Chen, Naifei Chen, Wei Song, Yongfei Zhang, Zheng Lv, Fujun Han, Yuguang Zhao, Wei Li, Jiuwei Cui

**Affiliations:** Cancer Center, The First Hospital of Jilin University, Changchun 130021, Jilin, China

## Abstract

**Objective:**

We aimed to retrospectively analyze the predictors of immune checkpoint inhibitors (ICIs)-efficacy in patients with advanced pancancer who were treated with various ICIs in the real world and focused on the correlation between ICIs-efficacy and immune-related adverse events (irAEs).

**Methods:**

We retrospectively analyzed data from 103 patients with advanced pancancer treated receiving various ICIs in the First Hospital of Jilin University from January 1, 2016 to August 1, 2020. Survival probabilities of progression-free survival (PFS) and overall survival (OS) were estimated using Kaplan–Meier curves and log-rank tests and the multivariate Cox proportional hazards model. Receiver-operating characteristic curve was used to determine a cutoff value for parameters and area under the curve. Correlations between the two variables were analyzed by logistic regression.

**Results:**

All patients were analyzed for survival predictors of OS, while 87 of 103 patients experienced evaluable disease progression of immunotherapy and were included in the analysis of predictors of PFS. First, we found that lower platelet (cutoff = 201.5 × 10^9^/L) and lactate dehydrogenase (LDH) (cutoff = 227 U/L) were independently associated with significantly improved PFS, while lower platelet-lymphocyte ratio (cutoff = 206.5), absolute monocyte count (cutoff = 0.62 × 10^9^/L), and LDH (cutoff = 194.5 U/L) were significantly and independently associated with better OS. In the analysis of the immune cell subgroup, a lower absolute countof CD8^+^CD28^−^suppressor T cells was an independent factor associated with better PFS (6.60 vs.4.13 months (mo), hazard ratios (HR) = 3.17, *p* = 0.0038), and OS (29.4 vs. 9.57 mo, HR = 3.05, *p* = 0.03). Second, the results of the analysis for irAEs showed that patients with any grade irAEs had higher objective response rate (30% vs. 10%, HR = 4.34, *p* = 0.009), disease control rate (69.7% vs. 50%, HR = 2.3, *p* = 0.028), PFS (8.37 vs. 3.77 mo, HR = 2.02, *p* = 0.0038), and OS (24.77 vs.13.83 mo, HR = 1.84, *p* = 0.024). Moreover, the groups with irAEs of grade ≥2 and with “multi-site” irAEs had significantly better PFS and OS (*p* < 0.05) compared with the other groups. We also proved that endocrine irAEs (usually thyroid dysfunction) were significantly associated with better mPFS (*p* = 0.01), and hepatic irAEs were significantly associated with better mOS (*p* = 0.023).

**Conclusions:**

This retrospective study explored the availability and effectiveness of some cost-effective and readily available blood biochemical parameters in routine clinical practice to predict the ICIs-efficacy and demonstrated the predictive role of different categories of irAEs on efficacy.

## 1. Introduction

As a novel class of antitumor drugs, immune checkpoint inhibitors (ICIs) have shown durable and significant efficacy in the treatment of a variety of malignant tumors [1–4]. At present, many ICIs have been approved for clinical practice, including anticytotoxic T lymphocyte-associated antigen-4 (CTLA-4) monoclonal antibody, ipilimumab, antiprogrammed cell death protein-1(PD-1) monoclonal antibody, nivolumab, pembrolizumab, and antiprogrammed cell death ligand-1(PD-L1) monoclonal antibody, atezolizumab; in addition, a variety of anti-PD-(L)1 drugs in China are being continuously developed and gradually applied in clinical trials and practice, such as toripalimab (JS001), sintilimab (IBI308), and camrelizumab (SHR-1210). However, only a subset of patients experience clinical benefit from these therapies; for example, in melanoma that responds well to ICIs, the disease response rate accounts for only 20% of ipilimumab treatment and 35%–60% of anti-PD-(L)1 treatment [5–7]. With the advent of alternative therapies such as combination targeted therapy or other immunotherapies, it will be particularly important to identify patients who may experience clinical benefit. Although a number of studies are identifying biomarkers that can be used to predict treatment outcomes [8, 9], such as PD-L1, tumor mutation burden (TMB), and microsatellite instability-high (MSI-H), these are far from being clarified, and many patients in clinical practice do not test routinely. Therefore, screening clinically convenient and available markers is essential for judging patient benefit before treatment. So far, reliable laboratory parameters have not been established in daily clinical routine to predict the clinical outcome after treatment with ICIs, but some studies have shown that such convenient, practical, and cost-effective indicators may be helpful in selecting patients who may benefit, while guiding those with a low chance of alternative treatment. On the other hand, due to the specific targets and mechanisms of action of ICIs, they may attack normal tissues and organs of the human body while activating the immune system, causing autoimmune and inflammatory effects at the corresponding sites, known as immune-related adverse events (irAEs) [10]. Many patients treated with ICIs experience irAEs, and severe cases can affect the treatment process and efficacy response. The basis of irAEs is the activation of abnormal autoimmune T lymphocytes, indicating a possible association between their occurrence and clinical benefit. Although a number of recent studies have reported this view [11–13], data on the impact of irAEs on long-term survival outcomes in clinical practice are conflicting and have not been clarified. Based on this, this retrospective study aimed to comprehensively analyze the convenient and available indicators that can predict the efficacy of ICIs in patients with advanced pancancer who were treated with multiple types of ICIs in the real world and focused on the correlation between ICIs-efficacy and irAEs that are currently of great concern to clinicians and clinical outcomes.

## 2. Patients and Methods

This study was a retrospective analysis approved by the Institutional Review Board (IRB) of the First Hospital of Jilin University to collect information from all patients with advanced pancancer who received ICIs in the First Hospital of Jilin University from January 1, 2016 to August 2, 2020. A detailed manual chart review was performed for each patient to record clinical data. Baseline characteristic data included age, gender, ECOG PS score, body mass index (BMI), smoking status, tumor type and stage, distant metastasis, previous treatment, number of treatment lines, immunotherapy regimens, available laboratory tests (including blood count and related ratio parameters, baseline lactate dehydrogenase (LDH) level, thyroid function indicators, and partially available venous immune cell count), and imaging examination. Clinical response to treatment was classified as complete response (CR), partial response (PR), stable disease (SD), or progressive disease (PD) according to Response Evaluation Criteria in Solid Tumors Version 1.1 (RECIST v1.1) and was measured by one imaging physician and two oncologists. Although the timing of CT scan analysis varies among tumor types, it is usually performed every 6–10 weeks. Objective response rate (ORR) is based on the combination of CR and PR, and disease control rate (DCR) is based on the combination of CR, PR, and SD. All patients were followed up for progression and survival until death or loss to follow-up. Overall survival (OS) was defined as the time from treatment initiation to death from any cause, censoring patients who are still alive at the date of follow-up. Progression-free survival (PFS) was defined as the time from treatment initiation to progressive disease or death from any cause, whichever came first. Patients who survived without disease progression were censored at the follow-up date. In addition, irAEs assessed by patients every 4 weeks were recorded throughout the treatment, which are defined as adverse events (AEs) that are related to immunotherapeutic drugs during immunotherapy, and have a potential immunological basis, including the skin, endocrine, pulmonary, gastrointestinal, hepatic, neurological, hematological, and other rare AEs. Occurring only one of these events is defined as “single-site” irAE, and two or more events are defined as “multi-site” irAEs. Clinical severity of irAEs was graded according to Common Terminology Criteria Adverse Events V4.0. In this study, serious irAEs were defined as irAEs of grades 3-4 or any grade irAEs leading to discontinuation of medication. The primary endpoint was OS, and secondary endpoints were PFS and irAEs.

Data were summarized using basic descriptive statistics. The cutoff date for survival analysis was 01 August 2020. Survival probabilities were estimated using Kaplan–Meier curves and log-rank tests, and based on that, all covariates with *p*-values less than 0.1 were included in a multivariate Cox proportional hazards model to estimate hazard ratios (HRs) and 95% confidence interval (CI). Receiver-operating characteristic (ROC) curve was applied to calculate cutoff values for the laboratory parameters and area under the curves [14]. The cutoff point was determined using Youden's index. For the two time variables of OS and PFS, in the ROC curve, after considering most of the patient survival data, we used whether a 12-month PFS or an 18-month OS was reached as the outcome status, respectively, to ensure that all patients were followed up for a consistent period of time. Throughout the analyses, all *p* values were based on a 2-sided hypothesis, and those less than 0.05 were considered statistically significant. When assessing the predictor model, Hosmer–Leme show the goodness-of-fit test was used to assess the completeness and predictive accuracy of the model. Correlations between the two variables were analyzed by logistic regression. We performed all statistical analyses using Medcalc version18.6 and SPSS 22.0 software.

## 3. Result

### 3.1. Patient Characteristics and Treatment

In this retrospective study, 103 patients with advanced tumors (stage IV or stage III tumors that progressed after treatment) who received ICIs were included in the final analysis. The study included lung cancer, 51 patients, melanoma, 15 patients, esophageal cancer, 11 patients, liver cancer, 9 patients, urothelial cancer, 7 patients, gastric cancer, 5 patients, and other types of tumors (including hypopharyngeal carcinoma, nasopharyngeal carcinoma, colon cancer, pancreatic cancer, and orbital malignant tumor, 1 patients, respectively). 80 patients (77.67%) received anti-PD-1 therapy, including 16 patients treated with nivolumab, 12 patients treated with pembrolizumab, and 52 patients treated with anti-PD-1 drugs made in China, including toripalimab (JS001), sintilimab (IBI308), tislelizumab (BGB-A317), and camrelizumab (SHR-1210), 13 patients (12.62%) received anti-PD-L1 therapy (atezolizumab), and 10 patients (9.71%) received nivolumab plus ipilimumab. In the overall population, PFS and OS were 4.1 (0.6–33.5) months and 10.0 (0.6–46.2) months, respectively. Our study required patients to be observed for at least 4–6 weeks for imaging response evaluation; thereby, 10 patients with AEs or other reasons who could not be evaluated or had insufficient time for evaluation were excluded. According to RECIST v1.1 criteria, 16 (17.20%) patients had PR, 37 patients (39.78%) had SD, and 40 patients (43.01%) had PD leaving no CR patients. The overall ORR was 17.20%, and the DCR was 56.99%.

### 3.2. Analysis of Predictors of Survival

#### 3.2.1. Analysis of Predictors of PFS

**Figure 1:**
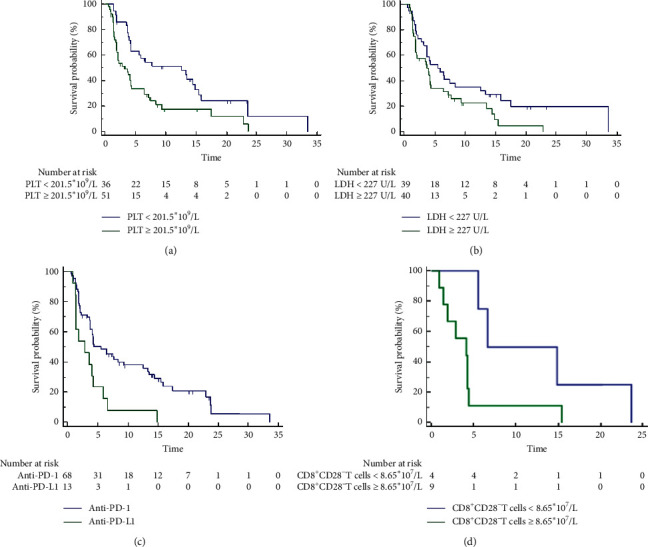
The analysis of progression-free survival (PFS) between different groups. (a) Platelet (PLT); (b) lactate dehydrogenase (LDH); (c) different treatment regimens; (d) absolute count of CD8^+^CD28^−^ T cell.

The baseline characteristics of the patients are described in detail in Table 1. In this study, 87 of 103 patients experienced evaluable disease progression of immunotherapy. We observed and analyzed the clinical characteristics and baseline blood test parameters of these patients. The results of Kaplan–Meier analysis showed that BMI, bone metastasis, absolute lymphocyte count (ALC), platelet (PLT) (Figure 1(a)), white blood cell (WBC), and lactate dehydrogenase (LDH) (Figure 1(b)) were related factors of PFS (Table 2). Multivariate analysis showed that low PLT (cutoff = 201.5 × 10^9^/L) and LDH (cutoff = 227 U/L) were independent factors. Other factors, such as gender, age, ECOG score, smoking status, distant metastasis, and number of treatment lines, were not significantly correlated with PFS. As for different treatment regimens, the efficacy of anti-PD-1 was superior to anti-PD-L1 (mPFS: 5.53 mo (95% CI: 3.77–12.53) vs. 2.83 mo (95% CI: 1.37–4.27), HR = 2.44, 95% CI: 1.06–5.63, *p* = 0.003) (Figure 1(c)). Subsequent analysis of 14 patients with immune cell parameters showed that a lower absolute count of CD8^+^CD28^−^suppressor T cells was an independent factor associated with PFS (cutoff = 8.65 × 10^7^/L, mPFS: 6.60 mo (5.530–23.670) vs. 4.13 mo (1.9–4.27), HR = 2.89, 0.96–8.63, 95% CI: 1.07–9.40, *p* = 0.044) (Figure 1(d)).

#### 3.2.2. Analysis of Predictors of OS

**Figure 2:**
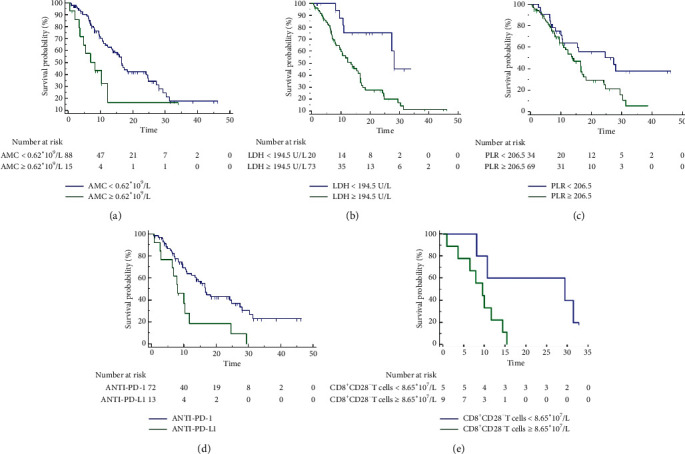
The analysis of overall survival (OS) between different groups. (a) Absolute monocyte count (AMC); (b) lactate dehydrogenase (LDH); (c) platelet-lymphocyte ratio (PLR); (d) different treatment regimens; (e) absolute count of CD8^+^CD28^−^ T cells.

In this study, all patients were analyzed for survival predictors of OS. The results of Kaplan–Meier analysis showed that absolute monocyte count (AMC) (Figure 2(a)), LDH (Figure 2(b)), and platelet-lymphocyte ratio (PLR) (Figure 2(c)) were predictors of OS, while other factors such as gender, age, ECOG score, BMI, smoking status, tumor type, distant metastasis, and number of treatment lines had no significant correlation with OS (Table 3). The results of multivariate analysis confirmed that low PLR (cutoff = 206.5), low AMC (cutoff = 0.62 × 10^9^/L), and low LDH (cutoff = 194.5 U/L) were independent factors associated with OS. The chi-square of this multivariate prediction model was 29.481 with a significance level (*p* < 0.0001), which considered that this model could better predict OS. As for different treatment regimens, the efficacy of anti-PD-1 was superior to anti-PD-L1 (mOS: 16.70 mo (95% CI: 14.57–18.83) vs. 8.07 mo (95% CI: 4.62–11.51), HR = 2.33, 95% CI: 1.03–5.31, *p* = 0.005) (Figure 2(d)). Subsequent analysis of 14 patients with immune cell parameters showed that a lower absolute count of CD8^+^CD28^−^suppressor T cells(cutoff = 8.65 × 10^7^/L, mOS: 29.4 mo (8.07–31.37) vs. 9.57 mo (6.47–11.63), HR = 3.05, 95% CI: 1.01–9.15, *p* = 0.03) was a significant factor associated with OS (Figure 2(e)).

### 3.3. Characteristics and Management of irAEs

Among 103 patients with advanced tumors, 41 patients (39.8%), 23 patients (22.33%), 14 patients (13.59%), and 10 patients (9.71%) experienced any grade irAEs, irAEs of grade ≥2, serious irAEs, and irAEs of grade ≥3, respectively. In patients with irAEs of any grade, there were 20 patients with “multi-site” irAEs (number: 2; range, 2–7) and 21 patients with “single-site” irAEs. The more common irAEs were endocrine AEs (18.45% (19/103)), skin response (12.62% (13/103)), immune-related liver injury (12.62% (13/103)), and hematological AEs (5.83% (6/103)). The safety of different drugs was different. The incidence of irAEs of anti-PD-1 was higher (37.5% (30/80)). Although most AEs were in grades 1-2, still 11.25% (9/80) experienced grades 3-4 or irAEs leading to drug withdrawal, while for anti-PD-L1, the incidence of irAEs was 7.69% (1/13), without serious irAEs. The incidence of any grade irAEs and serious irAEs was higher in the combination of double ICIs, 80% (8/10) and 50% (5/10), respectively. All patients in our study were managed for irAEs according to the Clinical Practice Guideline [15]. For patients with rash and pruritus of grades 1-2, loratadine was used for antiallergic treatment; for endocrine events of grades 2-3, such as diabetes and hypothyroidism, the drug is still applied after hypoglycemic therapy and hormone replacement therapy are used to maintain stable condition. All patients with immune-related liver injury of grades 3-4 were improved after intravenous steroid therapy combined with hepatoprotective, enzyme reduction, and symptomatic and supportive treatment, among which, patients with grade 4 events permanently discontinued the drug, and some patients chose to continue drug treatment after liver function recovery for grade 3 events. For patients with grade 2 pancreatitis and grade 4 lipase elevation, the drug was permanently discontinued and gradually improved after intravenous methylprednisolone treatment. One patient who experienced deficiency of factor VIII improved after discontinuation of the drug and supplementation of coagulation factors, transfusion of plasma, or cryoprecipitate. Patients with interstitial pneumonia of grade 2 were improved after steroid therapy, and immunotherapy was continued. No serious irAEs were found to develop into refractory.

### 3.4. Correlation Analysis between irAEs and ICIs-Efficacy

#### 3.4.1. Correlation Analysis between irAEs and Imaging Response Evaluation

In this study, we set that the evaluation of ICIs efficacy should be observed for at least 4–6 courses after administration. Of the 93 evaluable patients, 33 patients experienced irAEs, with 30.30% PR, 39.39% SD, and 30.30% PD, and none of patients had CR; 60 patients did not experience irAEs, with 10.00% PR, 40.00% SD, and 50.00% PD, and none of patients had CR. In the correlation analysis, ORR in the irAE group was significantly higher than that in the non-irAE group (ORR: 30% vs. 10%, HR = 4.34, 95% CI: 1.43–12.6, *p* = 0.009). DCR was also significantly higher in the irAE group than in the non-irAE group (DCR: 69.7% vs. 50%; HR = 2.3, 95% CI: 1.10–4.83, *p* = 0.028) (Table 4).

#### 3.4.2. Correlation Analysis between irAEs and Survival Outcomes

We first compared the effect of baseline characteristics (age, gender, ECOG score, BMI, smoking status, distant metastasis, and treatment lines) on survival outcomes and found that there was no significant correlation and that only irAEs were independent factors associated with survival outcomes (Table 5). Next, we performed survival analysis separately for irAEs.

**Figure 3:**
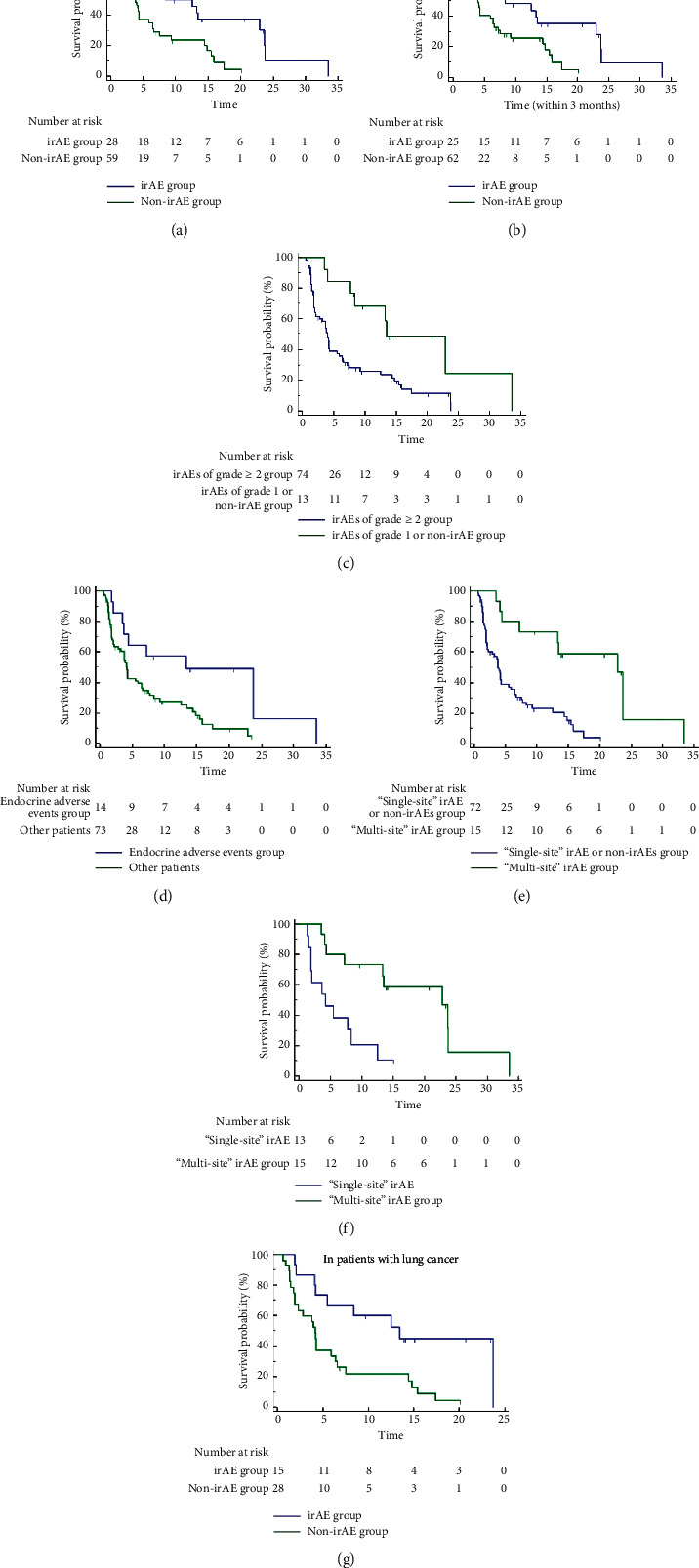
The correlation of irAEs with progression-free survival (PFS). (a) In total patients, the irAE group and non-irAE group. (b) In total patients, the irAE group and other patients group within 3 months. (c) In total patients, irAEs of the grade ≥2 group and irAEs of the grade 1 or non-irAE group. (d) In total patients, the endocrine AEs group and other patients group. (e) In total patients, the “single-site” irAE or non-irAEs group and “multi-site” irAE group. (f) In the irAE subgroup, the “single-site” irAE group and “multi-site” irAE group. (g) In patients with lung cancer, the irAE group and non-irAE groups.

**Figure 4:**
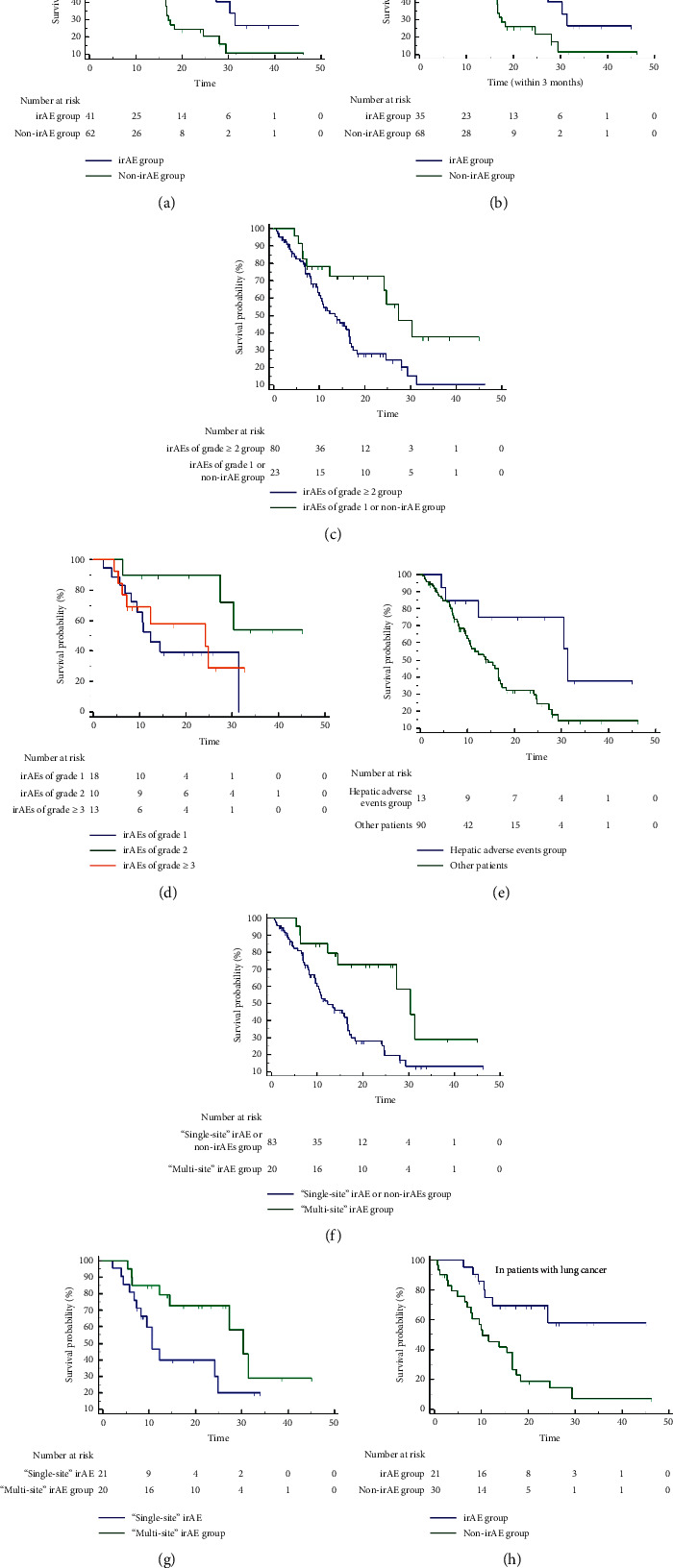
The correlation of irAEs with overall survival (OS). (a) In total patients, the irAE group and non-irAE group. (b) In total patients, the irAE group and other patients group within 3 months. (c) In total patients, irAEs of the grade ≥2 group and irAEs of the grade 1 or non-irAE group. (d) In the irAE subgroup, irAEs of grades 1, 2, and ≥3. (e) In total patients, the hepatic AEs group and other patients group. (f) In total patients, the “single-site” irAE or non-irAEs group and “multi-site” irAE group. (g) In the irAE subgroup, the “single-site” irAE group and “multi-site” irAE group. (h) In patients with lung cancer, the irAE group and non-irAE groups.

(i) Survival Analysis of irAEs and PFS
  1. In total patients, there was a significant difference in PFS between the irAE group with non-irAE (mPFS: 8.37 mo (95% CI: 4.37–22.9) vs. 3.77 mo (95% CI: 2.1–4.27), HR = 2.02, 95% CI: 1.25–3.26, *p* = 0.0038; 12-month PFS rate: 43% vs. 12%, HR = 0.18, 95% CI: 0.06–0.53, *p* = 0.002) (Figure 3(a)). In addition, PFS was significantly higher in patients with any grade irAEs within 3 months compared with other patients (mPFS: 8.37 mo (95% CI: 4.1–22.9) vs. 3.97 mo (95% CI: 2.27–6.43), HR = 1.79, 95% CI: 1.10–2.90, *p* = 0.018) (Figure 3(b)).
  2. In all patients, PFS was superior in the group with irAEs of grade ≥2 compared with the group with grade 1 only or non-irAE (mPFS: 13.47 mo (95% CI: 8.37–33.5) vs. 3.97 mo (95% CI: 2.83–5.53), HR = 0.38, 95% CI: 0.22–0.65, *p* = 0.005; 12-month PFS rate: 53.85% vs.16.22%, HR = 6.03, 95% CI: 1.72–21.11, *p* = 0.005). In patients with irAEs, there was only a difference trend in PFS between the patients with irAEs of grade ≥2 and those with grade 1 only (*p* = 0.072) (Figure 3(c)).
  3. In total patients, there was a significant difference in PFS with and without any grade endocrine AEs (mPFS: 13.3 mo (95% CI: 3.67–23.73) vs. 4.13 mo (95% CI: 3.27–6.43), HR = 2.19, 95% CI: 1.28–3.74, *p* = 0.01; 12-month PFS rate: 58.33% vs. 16%, HR = 0.20, 95% CI: 0.06–0.67, *p* = 0.010) (Figure 3(d))
  4. In all patients, the difference of PFS between patients with “single-site” irAE or non-irAEs and “multi-site” irAEs of any grade was significant (mPFS: 3.77 mo (95% CI: 2.27–5.53) vs. 22.9 mo (95% CI: 13.3–23.73), HR = 0.33, 95% CI: 0.20–0.54, *p* = 0.0002; 12-month PFS rate: 12.5% vs. 66.67%, HR = 14.0, 95% CI: 3.89–50.39, *p* < 0.001) (Figure 3(e))
  5. In the irAE subgroup, there was a significant difference in PFS between groups of “single-site” and “multi-site” irAE (mPFS: 4.233 mo (95% CI: 1.867–8.367) vs. 22.9 mo (95% CI: 13.467–23.733), HR = 0.31, 95% CI: 0.12–0.83, *p* = 0.0024; 12-month PFS rate: 15.38% vs. 66.67%, HR = 11.0, 95% CI: 1.73–69.96, *p* = 0.011) (Figure 3(f))
  6. Survival analysis of PFS of 43 patients with advanced lung cancer showed that there was a significant difference in PFS between the irAE group and non-irAE group (mPFS: 13.47 mo (95% CI: 5.53–23.73) vs. 4.13 mo (95%CI: 1.9–6.43), HR = 2.66, 95% CI: 1.36–5.20, *p* = 0.007; 12-month PFS rate: 53.33% vs. 17.86%, HR = 0.19, 95% CI: 0.47–0.77, *p* = 0.02) (Figure 3(g))
(ii) Survival Analysis of irAEs and OS
  1. In total patients, there was a significant difference in OS between the irAE group and non-irAE (mOS: 24.77 mo (95% CI: 10.68–38.86) vs. 13.83 mo (95% CI: 8.16–19.51), HR = 1.84, 95% CI: 1.09–3.09, *p* = 0.024; 18-month OS rate: 37% vs. 16%, HR = 0.33, 95% CI: 0.13–0.84, *p* = 0.020) (Figure 4(a)). In addition, OS was significantly higher in patients with any grade irAEs within 3 months compared with other patients (mOS: 24.77 mo (95% CI: 12.3–31.37) vs. 13.83 mo (95% CI: 9.63–16.7), HR = 1.71, 95% CI: 1.03–2.86, *p* = 0.046) (Figure 4(b)).
  2. In all patients, OS of patients with irAEs of grade ≥2 was superior to that of patients with grade 1 only or non-irAE (mOS: 27.33 mo (95% CI: 19.45–35.22) vs. 13.47 mo (95% CI: 8.36–18.57), HR = 0.43, 95% CI: 0.25–0.75, *p* = 0.012; 18-month OS rate: 43.48% vs. 18.75%, HR = 3.33, 95% CI: 1.23–9.04, *p* = 0.018) (Figure 4(c)). In the patients with irAEs, there was only a difference trend in OS between patients with irAEs of grade ≥2 and those with grade 1 only (*p* = 0.077), and there was a significant difference in OS between patients with irAEs of grade ≥2 and other grades of irAEs (grade 2 vs. grade 1: HR = 0.27, 95% CI: 0.10–0.78, *p* = 0.024; grade 2 vs. grade ≥3: *p* = 0.070) (Figure 4(d)).
  3. In total patients, there was a significant difference in OS with and without any grade of hepatic AEs (mOS: 31.37 mo (95% CI: 29.3–33.43) vs. 14.4 mo (95% CI: 9.42–19.38), HR = 2.72, 95% CI: 1.44–5.17, *p* = 0.023; 18-month OS rate: 53.85% vs. 20%, HR = 0.21, 95% CI: 0.06–0.71, *p* = 0.012) (Figure 4(e)).
  4. In all patients, the difference of OS between patients with “single-site” irAE or non-irAEs and “multi-site” irAEs of any grade was significant (mOS: 12.3 mo (95% CI: 9.97–16.63) vs. 30.37 mo (95% CI: 27.33–31.37), HR = 0.37, 95% CI: 0.21–0.64, *p* = 0.004; 18-month OS rate: 18.07% vs. 50%, HR = 4.53, 95% CI: 1.60–12.82, *p* = 0.004) (Figure 4(f)).
  5. In the irAE subgroup, there was a significant difference in OS between groups of “single-site” and “multi-site” irAE (mOS: 10.7 mo (95% CI: 7.34–14.06) vs. 30.37 mo (95% CI: 23.363–37.37), HR = 0.43, 95% CI: 0.18–1.01, *p* = 0.048; 18-month OS rate: 23.81% vs. 50%, *p* = 0.087) (Figure 4(g)).
  6. Survival analysis of PFS of 51 patients with advanced lung cancer showed that there was a significant difference in OS between the irAE group and non-irAE group (mOS: not reached vs. 10.33 mo (95% CI: 7.87–16.57), HR = 3.65, 95% CI: 1.8–7.38, *p* = 0.001; 18-month OS rate: 42.86% vs. 19.35%, *p* = 0.083) (Figure 4(h)).

## 4. Discussion

With the wide application and development of ICIs, improving their therapeutic efficacy has become an urgent problem to be solved and concerned. Several studies are identifying biomarkers that can be used to predict treatment outcomes [8, 9], such as PD-L1, TMB, and MSI-H, but these are far from being clarified and many patients in clinical practice do not test routinely. Therefore, screening clinically convenient and available markers is essential for judging the benefit of the patients before treatment. Based on this, this retrospective study analyzed the correlation between basic clinical characteristics, blood biochemical parameters, peripheral blood immune cells, and the efficacy of ICIs in patients with advanced pancancer and focused on exploring the correlation between different grades, types, and numbers of irAEs and clinical outcomes. This study did not find a significant correlation between clinical characteristics and the ICIs efficacy. Given that multiple blood cell and inflammatory factors are part of routine laboratory testing, they have been extensively examined as prognostic and predictive biomarkers. Studies suggest that complete blood counts may be markers of cancer inflammation and adaptive immune response and that laboratory parameters in clinical routine have the advantages of convenience, practicality, and economy in predicting clinical outcomes of patients treated with ICIs. Those have been reported in some previous studies, for example, higher pretreatment RLC and REC in patients treated with pembrolizumab [15], lower AMC [16], higher AEC [16], higher ALC [17], lower RLC [16] at baseline, and increased ALC and AEC during treatment [18] were associated with favorable survival outcomes. In terms of baseline blood and biochemical indicators, our study found that lower PLT (cutoff = 201.5 × 10^9^/L) and LDH (cutoff = 227 U/L) were independently associated with significantly improved PFS, while lower PLR (cutoff = 206.5), AMC (cutoff = 0.62 × 10^9^/L), and LDH (cutoff = 194.5 U/L) were significantly and independently associated with better OS. LDH are strong baseline biomarkers associated with prognosis, similar to those reported by others [16, 18, 19]. Especially in melanoma treated with ICIs, LDH and pattern of visceral metastases have been more recognized as prognostic factors and are incorporated into the AJCC staging classification [20]. In patients treated with ipilimumab [17, 18] and pembrolizumab [21], elevated baseline LDH has also been described as inversely associated with patient OS.

Our study also showed a negative effect of high AMC on patient survival outcomes, which is consistent with the results observed in the study by Martens et al. [16], but that study also examined the baseline frequency of Lin-CD14^+^HLADR^−^low myeloid-derived suppressor cells (MDSCs) using flow cytometry analysis, confirming that low baseline frequency CD14^+^ MDSCs are a powerful indicator of benefit, suggesting that both high AMC and CD14^+^ MDSCs could predict poor survival. Similar results were reported in two previous single-center studies [22], and a recent study by Gebhardt et al. [23] reported that clinical responses were associated with low levels of circulating MDSCs, providing a rationale for seeking therapeutic strategies aimed at depleting these cells. Moreover, Martens et al. [16] also established a combined prediction model for treatment outcome of ipilimumab defined based on six baseline biomarkers, low LDH, AMC, and MDSC and high AEC, Tregs, and RLC, and validated its better predictive power. Our study design and the available detection parameters were somewhat different from them; we only found the correlation between low LDH, AMC, and survival outcome but did not explore the correlation between high AEC and RLC on PFS or OS. This difference may be explained in part by the fact that AEC and RLC are significantly associated with irAEs of any grade or skin irAEs (rash or vitiligo) and that massive inflammation during the development of such irAEs can cause neutrophil expansion, which in turn significantly affects the immune environment. Other studies have similarly confirmed the association of blood cells and biochemical factors (e.g., LDH) with treatment outcomes [15, 24, 25]. In addition, PLTs are also part of the inflammatory response, and their increase can contribute to tumor growth, invasion, and factors of angiogenesis [26]. The association between PLTs and poor prognosis and shorter survival time has been established in several types of solid tumors, including the breast, lung, colon, gastric, and ovarian cancers [27], which is thought to result from the secretion of PLT-producing cytokines such as interleukin-6 (IL-6) by tumor cells [27]. With the recognition that low lymphocyte counts may also be associated with shorter survival [28], the PLR has also been gradually explored, which, as an inflammatory marker, has also been shown to be associated with poor prognosis in several malignancies. A systematic review evaluated the effect of PLR on survival outcomes in 20 studies including 12,754 patients and found that higher PLR was associated with significantly worse OS (HR = 1.87, *p* < 0.001), and PLR had a greater effect on OS in metastatic disease than in early disease [29]. These are consistent with our findings, suggesting that lower PLT and PLR are significantly associated with better survival outcomes; thus, further studies on the regulation and correlation of platelet-related parameters in daily practice are warranted in the future.

In addition, some patients with peripheral blood immune cell parameters were analyzed in this study, and the results showed that lower absolute count of CD8^+^CD28^−^suppressor T cellswas an independent factor associated with better PFS (6.60 vs. 4.13 mo, HR = 3.17, *p* = 0.0038) and OS (29.4 vs. 9.57 mo, HR = 3.05, *p* = 0.03). CD8^+^CD28^−^suppressor T cells are derived from the monoclonal expansion of T cells [30], which can act directly on APCs, resulting in the downregulation of the expression of costimulatory molecules and the induction of the upregulation of inhibitory receptors [31]. CD8^+^CD28^−^suppressor T lymphocytes are almost persistent and functional in human tumors and are able to inhibit both proliferation and cytotoxicity of T cells, with pathogenic relevance and significance for immunotherapy of cancer [32]. In in vitro experiments, CD8^+^CD28^−^ T cells inhibited the proliferation of CD4^+^effector T cells and their secretion of IFN-*γ*. A study showed that the elevated peripheral blood CD8^+^CD28^−^ T cells was associated with poorer prognosis for metastatic breast cancer, especially in patients with higher risk of progression in patients with first-line chemotherapy and higher risk of death more than second-line chemotherapy [33]. However, the role of immunosuppressive function of CD8^+^suppressorT cells in immune responses in patients receiving ICIs has not been studied. This study showed that patients with relatively higher CD8^+^CD28^−^ suppressor T cells were associated with worse PFS and OS, considering that it may be due to the inhibitory effect of CD8^+^CD28^−^ suppressor T cells on effector T cells. After the application of ICIs, it is not easy to activate the systemic immune system to produce response of the human body. However, unfortunately, due to the small sample size of patients with immune cells in this study, it needs to be interpreted with caution and only as an exploratory finding. Nevertheless, to our knowledge, our study discovered the role of CD8^+^CD28^−^ T cells in immune responses, and these data with suggestive significance are available only from a simple peripheral blood examination. Moreover, previous studies have only detected and analyzed CD8^+^T cells, and our study suggests whether they can be further divided into CD28^+^ and CD28^+^ T cells in the future to more accurately understand their role in the body's immune response. At present, it is unclear whether these proposed baseline blood immune-inflammation-related markers have a specific predictive impact on outcomes of patients after treatment with ICIs, but this provides information and reference for randomized controlled trials. Future prospective studies with larger sample sizes are needed to further verify the power of these parameters.

The incidence of irAEs of any grade observed in this study was 39.8%, mainly mild and transient, and the incidence of serious irAEs and irAEs ≥ grade 3 was 13.59% and 9.7% respectively. Generally, ICIs were well tolerated, and the overall toxicity profile was consistent with previous studies [6, 34, 35]. The most common AEs were endocrine disorders and thyroid disorders, followed by immune-mediated skin-related diseases (mainly rash with dry skin and itching) and immune-related liver injury, a large proportion of which is considered to be related to immune combinations. The serious irAEs in this study were all improved after intravenous corticosteroids and did not develop into refractory. No treatment-related deaths were observed. Given the similar immunological basis between immune-related toxicity and the clinical benefit of ICIs, several recent retrospective studies have investigated the correlation between irAEs and the efficacy of immunotherapy, especially in lung cancer and melanoma, which have shown positive results, such as data from nivolumab-treated nonsmall cell lung cancer (NSCLC) patients showed that ORR and PFS were significantly better in patients with irAEs than in those without [34]; however, in a meta-analysis of 4 prospective studies in different cancers, any grade irAEs were associated with higher overall response rates, but no effect on PFS was detected [34]. Rogado et al. [36] found that PFS was significantly shorter (3 vs. 10 mo, HR = 2.2, *p* = 0.016) in patients without any grade irAEs in a study of 153 patients treated with anti-PD-(L)1, but the mOS was not significant. Therefore, it remains unknown whether the occurrence of irAEs can indicate treatment response and translates into better survival outcomes in clinical practice. Based on that, our study conducted a retrospective analysis of 103 patients in the real world, further emphasizing the importance of irAEs in predicting survival outcomes. The overall results showed that patients with any grade irAEs had higher ORR (30% vs. 10%, HR = 4.34, *p* = 0.009), DCR (69.7% vs. 50%, HR = 2.3, *p* = 0.028), mPFS (8.37 vs. 3.77 mo, HR = 2.02, *p* = 0.0038), and mOS (24.77 vs. 13.83 mo, HR = 1.84, *p* = 0.024), indicating a strong association between the occurrence of irAEs and better treatment efficacy. The emergence of irAEs can translate into a better clinical survival benefit. In addition, several studies have reported the association between irAEs and long-term survival outcomes in patients treated with different ICIs in different cancer types, but the results are conflicting [34, 37–39]. In this study, the correlation between any grade irAEs and survival outcome in different cancer types was also analyzed, but there was no correlation in common tumors such as melanoma and bladder cancer (which may be due to the small samples). Only in lung cancer patients with a large sample size, the occurrence of any grade irAEs was significantly associated with better mPFS (13.47 vs. 4.13 mo, HR = 2.66, *p* = 0.007) and mOS (not reached vs. 10.33 mo, HR = 3.65, *p* = 0.001), which is consistent with most other retrospective studies on NSCLC, including some that appropriately consider the time-varying nature of the occurrence of irAEs [11, 40, 41]. We considered that overall heterogeneity of the results was related to the types of ICIs, unique characteristics of patients, and the heterogeneity of tumor types.

Theoretically, excessive activation of free T cells can cause higher grade irAEs and may also enhance the antitumor immune response, which leads to a further analysis of the effect of different grades of irAEs on clinical outcomes in our study. The results showed that among all patients, the group with irAEs of grade ≥2 had better PFS and OS compared with the group with grade 1 only or non-irAE, while in the irAE subgroup, the patients with irAEs of grade ≥2 tended to have better PFS and OS than those with grade 1. We further subdivided patients with irAEs into three categories by grade as grade 1, grade 2, and grade 3 or higher and found that the OS of patients with grade 2 irAEs was not only significantly longer than that of patients with grade 1 irAEs (HR = 0.27, *p* = 0.024) but also longer than that of patients with irAEs of grade ≥3 (*p* = 0.070). Evidence suggests that the occurrence of irAEs of grade 1 has little to do with the activation of the immune system, while grade 2 irAEs (usually without serious sequelae) may predict the activation of the immune system and better efficacy with a positive predictive effect. Conversely, more severe grades of irAEs are unlikely to indicate better clinical outcomes because the balance between advantages or disadvantages of irAEs themselves depends on their severity. In addition, although irAEs tended to be organ-specific, the incidence of “single-site” irAEs was essentially the same as that of “multi-site” irAEs in patients with various types of malignancies in this study. The number of irAEs may indicate different degrees of activation of the body's immune system and be associated with the efficacy of immunotherapy. A report of NSCLC showed that patients with “multi-site” irAEs survived longer than patients with “single-site” irAEs only [42]. However, Maillet et al. [11] showed that OS was more associated with the occurrence of “single-site” irAEs (*p* < 0.0001). Therefore, it is not clear whether the number of irAEs can differentially affect clinical outcomes in patients with tumors treated with ICIs. Based on the occurrence of irAEs reflecting immune system activation, we hypothesized that the number of irAEs reflects the degree and intensity of the activation of the immune system, that is, stronger immune activation of ICIs can induce irAEs at multiple sites and further produce a better antitumor effect. Eventually, we proved this hypothesis, and the findings showed that PFS and OS in patients with “multi-site” irAEs were significantly better than those in patients with “single-site” irAEs or non-irAEs in the overall patient population (mPFS: 22.9 vs. 3.77 mo, HR = 0.33, *p* = 0.0002; mOS: 30.37 vs.12.3 mo, HR = 0.37, *p* = 0.004), while the same phenomenon was observed in patients who had experienced irAEs. Therefore, it is conceivable that patients with a single irAE are more likely to develop a specific irAE, but it does not reflect the activation of the immune system after ICI administration, while the occurrence of multiple irAEs may indicate that ICIs induce a stronger and wider systemic immune activation. In addition, ICIs can target not only tumor-specific T cells but also other T cells, which may cause unexpected activation of nontumor-specific T cells, leading to irAEs in different organs [43]. Therefore, the occurrence of “multi-site” irAEs is more likely to be a predictive biomarker of improved efficacy. In addition, the type of organ-specific irAEs was also associated with ICIs efficacy. In this study, endocrine irAEs (usually thyroid dysfunction) were significantly associated with better mPFS (13.3 vs. 4.13 mo, HR = 2.19, *p* = 0.01), and hepatic irAE events were significantly associated with better mOS (31.37 vs. 14.4 months, HR = 2.72, *p* = 0.023), but not all irAEs had the same effect on clinical outcomes. It was speculated that the predictive function may depend on the system/organ involved. A recent retrospective study in 134 patients with NSCLC who were treated with nivolumab showed a statistically significant association of any grade irAEs, skin irAEs, and endocrine irAEs with prolonged PFS, whereas only any grade irAEs and skin irAEs were associated with prolonged OS [44]. As mentioned previously, the basic mechanisms of tumor responses during immunotherapy are the same as those for irAEs, which depend on the activation of “tissue-specific” immune self-responses via T cell-and B cell-mediated pathways. Thus, underlying “tissue-specific” autoimmunity is not only associated with treatment but also with patient characteristics.

This study is a small, retrospective, and non-randomized study in a single center with some limitations. First, the sample size of observed patients is small and may be affected by the intrinsic selection bias caused by the retrospective study itself. Second, the definition criteria for irAEs are different in different studies, and the determination of irAEs in clinical practice may be somewhat subjective, some of which are only diagnosed by exclusion. Therefore, the results in this study should be interpreted objectively and with caution. Third, other studies have reported that some biomarkers related to ICIs efficacy based on gene expression and immune microenvironment, such as PD-L1, TMB, and MSI-H, may more accurately predict efficacy and guide clinical decision-making. However, due to the lack of tissue samples in most patients, this study failed to assess these biomarkers. In addition, although our study discussed some simple and conveniently obtained predictive markers for the efficacy of immunotherapy, it is far from sufficient in mining the important role of liquid biopsy in cancer immunity. Currently, there is increasing interest in the relationship between cancer and immune heterogeneity. Liquid biopsy based on isolation and analysis of tumor-derived or tumor-associated components circulating in the blood allows longitudinal evaluation of cancer progression, can serve as a potential tool to capture tumor heterogeneity in metastatic cancer patients, and also help identify biomarkers that influence treatment decisions [45]. Through the analysis of temporal and spatial heterogeneity of circulating tumor cells (CTCs), more unique insights into tumor heterogeneity may be obtained than tissue biopsies. For example, immune checkpoint biomarkers have been analyzed on CTCs, especially in metastatic NSCLC and breast cancer patients, showing high interindividual heterogeneity of PD-L1 expression [46]. The number, subsets, and molecular characteristics of circulating leukocytes have been analyzed in cancer patients as prognostic and predictive biomarkers and can serve as powerful tools to detect the immunological cancer heterogeneity. For example, T cell receptor (TCR) profiling and surface immunoprofiling of circulating leukocytes are emerging powerful tools to measure T cell heterogeneity and immunogenic neoantigen burden in infiltrating tumor samples [45], but the lack of standardized protocols and the requirement of very sophisticated and expensive technologies to perform such analyses limit its use in the clinical setting. In addition, the quantitative and qualitative analyses of tumor-derived circulating nucleic acids, such as circulating tumor DNA (ctDNA), can provide relevant clinical information on tumor burden, stage, vascularity, and treatment response; circulating RNA may help clinicians track treatment response or drug resistance and reflect tumor heterogeneity. Due to the diversity of identifiable analytes and the repeatability of detection, liquid biopsies represent an accessible tool to decode spatial and temporal tumor heterogeneity. In the future, it may significantly and noninvasively contribute to the clinical management and treatment decisions for cancer patients in the era of precision medicine.

Moreover, tumor microenvironment (TME), a complex ecosystem, also represents an additional source of intratumoral heterogeneity. The TME might deeply shape the cancer milieu by inducing a permissive niche. A study showed that lymph node-positive patients with Wntsignaling pathway overexpression along with immune suppressive microenvironment enrichment may show immune escape, enhanced tumor invasiveness, epithelial-mesenchymal transition, drug resistance, and metastatic potential [47]. Halting these vicious cycle hold the promise to more efficiently employ immune checkpoint inhibitors. Therefore, a full understanding of tumor and immune heterogeneity, as well as the role of the TME, may add important findings to the immune landscape of malignancies and their implications and may lead to a deeper exploration of liquid biopsy-based biomarkers of immunotherapy.

## 5. Conclusion

Overall, this study reported data from real-world patients with unselected pancancer treated with ICIs. We explored the availability and effectiveness of some cost-effective and most readily available blood biochemical parameters and markers of immune inflammatory in routine clinical practice to predict the ICI efficacy and survival outcomes of patients, being an important clinical research value. Moreover, we then fully and comprehensively reported the correlation between ICI efficacy and the occurrence, number, and severity of irAEs, emphasizing that maintaining ICI treatment in patients with irAEs should be a priority. Evidence of a correlation between safety and treatment efficacy may ultimately be integrated into clinical decisions for patients who experience irAEs, to fully assess the patient risk-benefit ratio based on the type and severity of irAEs and disease response. Carefully manage treatment-related toxicities to maximize clinical benefit for patients treated with immunotherapy. Although these findings need to be further validated in large-scale retrospective and prospective, randomized controlled studies, our study provides important information and reference value for predictors of immunotherapy and prognosis stratification of patients in future studies.

## Figures and Tables

**Table 1:** Baseline characteristics of the patients.

Variable		Number of patients/Value	Percentage (%)
Gender	Male	80	77.67
	Female	23	22.33
Age	Median	61	—
	Scope	24–77	
ECOG PS	0	17	16.50
	1	86	83.50
Body mass index	Mean value	23.4	—
	Scope	15.8–31.2	
Tumor type	Lung cancer	51	49.51
	Melanoma	15	14.56
	Esophageal cancer	11	10.68
	Liver cancer	9	8.74
	Urothelial carcinoma	7	6.80
	Gastric cancer	5	4.85
	Other types of tumors	5	4.85
Distant metastasis	No distant organ metastasis	65	63.10
	One or more distant metastatic cancers	38	36.90
Prior therapy	Chemotherapy	67	65.05
	Radiotherapy	22	21.36
	Targeted therapy	8	7.77
	Immunotherapy	3	2.91
	Other therapies (interferon therapy and interventional/radiofrequency ablation therapy)	9	8.74
Treatment lines	First-line	26	25.24
	Nonfirst-line	77	74.76
Treatment regimen	Anti-PD-1	80	77.67
	Anti-PD-L1	13	12.62
	Anti-PD-1 + anti-CTLA-4	10	9.71

—Represents no usability data.

**Table 2:** Analysis of predictors of PFS.

Variable	Kaplan–Meier analysis			Cox multivariate regression analysis		
	HR	95% CI	*p*	HR	95% CI	*p*
Age	0.80	0.49–1.29	0.34			
Sex	0.66	0.38–1.13	0.16			
ECOG PS	0.80	0.41–1.58	0.49			
BMI	0.59	0.36–0.96	0.030^*∗*^			
Smoking	0.91	0.571.48	0.71			
Distant metastasis	0.82	0.49–1.36	0.42			
Treatment lines	1.05	0.58–1.91	0.86			
Bone metastasis	0.38	0.13–1.07	0.0037^*∗*^	—	0.25–1.43	0.25
Treatment regimen (anti-PD-1/PD-L1)	2.44	1.06–5.63	0.003^*∗*^	2.85	1.47–5.52	0.0051^*∗*^
WBC (cutoff = 8.19 × 10^9^/L)	1.89	0.86–4.14	0.038^*∗*^	—	0.78–3.84	0.18
ALC (cutoff = 0.635 × 10^9^/L)	2.61	1.39–4.91	0.017^*∗*^	—	0.92–13.69	0.06
PLT (cutoff = 201.5 × 10^9^/L)	2.02	1.24–3.26	0.003^*∗*^	3.13	1.78–5.52	0.0008^*∗*^
LDH (cutoff = 227 U/L)	1.63	0.99–2.67	0.047^*∗*^	1.85	1.06–3.24	0.0315^*∗*^

^*∗*^Indicators with statistical significance; —Represents no usability data; WBC, white blood cell; LDH, lactate dehydrogenase; PLT, platelet; ALC, absolute lymphocyte count; PD-1, programmed cell death protein-1; PD-L1, programmed cell death ligand-1; BMI: body mass index.

**Table 3:** Analysis of predictors of OS.

Variable	Kaplan–Meier analysis			Cox multivariate regression analysis		
	HR	95% CI	*p*	HR	95% CI	*p*
Age	0.86	0.51–1.44	0.56			
Sex	0.78	0.41–1.46	0.46			
ECOG PS	1.04	0.51–2.09	0.92			
BMI	0.91	0.54–1.54	0.73			
Smoking	1.21	0.72–2.02	0.47			
Distant metastasis	0.69	0.40–1.19	0.16			
Treatment lines	0.97	0.53–1.79	0.86			
Bone metastasis	0.60	0.26–1.35	0.13			
Treatment regimen (anti-PD-1/PD-L1)	2.55	1.05–6.20	0.003^*∗*^	2.31	1.17–4.56	0.016^*∗*^
PLR (cutoff = 206.5)	1.84	1.09–3.10	0.032^*∗*^	2.12	1.08–4.15	0.028^*∗*^
LDH (cutoff = 194.5 U/L)	3.24	1.81–5.81	0.004^*∗*^	2.65	1.08–6.52	0.034^*∗*^
AMC (cutoff = 0.62 × 10^9^/L)	2.26	0.90–5.70	0.015^*∗*^	3.98	1.83–8.65	0.0005^*∗*^

^*∗*^Indicators with statistical significance; —Represents no usability data; AMC, absolute monocyte count; LDH, lactate dehydrogenase; PLR, plateletlymphocyte ratio; PD-1, programmed cell death protein-1; PD-L1, programmed cell death ligand-1; BMI, body mass index.

**Table 4:** The correlation of irAEs with response evaluation.

Population	Variable	Number	Percentage (%)	ORR (%)	DCR (%)	ORR (irAE and non-irAE)	DCR (irAE and non-irAE)
Total patient population (93 patients)	PR	16	17.20	17.2	56.99		
	SD	37	39.78				
	PD	40	43.01				
irAE group (33 patients)	PR	10	30.30	30	69.7	*p* = 0.009^*∗*^ (HR = 4.34, 95% CI: 1.43–12.6)	*p* = 0.028^*∗*^ (HR = 2.3, 95% CI: 1.10–4.83)
	SD	13	39.39				
	PD	10	30.30				
Non-irAE group (60 patients)	PR	6	10.00	10	50		
	SD	24	40.00				
	PD	30	50.00				

^∗^Indicators with statistical significance; ORR, objective response rate; DCR, disease control rate.

**Table 5:** Correlation analysis of baseline characteristics and irAEs with survival outcomes.

Survival outcomes	Variable	Kaplan–Meier analysis			Cox multivariate regression analysis		
		HR	95% CI	*p*	HR	95% CI	*p*
*Progression-free survival*	Age	0.80	0.49–1.29	0.34			
	Gender	0.66	0.38–1.13	0.16			
	ECOG PS	0.80	0.41–1.58	0.49			
	BMI	0.59	0.36–0.96	0.030^*∗*^	0.67	0.40–1.13	0.1371
	Smoking	0.91	0.571.48	0.71			
	Distant metastasis	0.82	0.49–1.36	0.42			
	Treatment lines	1.05	0.58–1.91	0.86			
	irAEs	2.02	1.25–3.26	0.0038^*∗*^	2.18	1.22–3.90	0.0087^*∗*^

*Overall survival*	Age	0.86	0.51–1.44	0.56			
	Gender	0.78	0.41–1.46	0.46			
	ECOG PS	1.04	0.51–2.09	0.92			
	BMI	0.91	0.54–1.54	0.73			
	Smoking	1.21	0.72–2.02	0.47			
	Distant metastasis	0.69	0.40–1.19	0.16			
	Treatment lines	0.97	0.53–1.79	0.86			
	irAEs	1.84	1.09–3.09	0.024^*∗*^			

^∗^Indicators with statistical significance; —Represents no usability data.

## Data Availability

The data used to support the findings of this study are included within this article and are available from the corresponding author upon request.

## Abbreviations

ICIs: Immune checkpoint inhibitors
CTLA-4: Cytotoxic T lymphocyte-associated antigen-4
PD-1: Programmed cell death protein-1
PD-L1: Programmed cell death ligand-1
irAEs: Immune-related adverse events
ROC curve: Receiver-operating characteristic curve
PFS: Progression-free survival
TMB: Tumor mutation burden
MSI-H: Microsatellite instability-high
OS: Overall survival
CR: Complete response
PR: Partial response
SD: Stable disease
PD: Progressive disease
RECIST v1.1: Response evaluation criteria in solid tumors version 1.1
ORR: Objective response rate
PLR: Platelet-lymphocyte ratio
MDSCs: Myeloid-derived suppressor cells
DCR: Disease control rate
RLC: Relative lymphocyte count
ALB: Albumin
AEC: Absolute eosinophil count
ALC: Absolute lymphocyte count
PLT: Platelet
WBC: White blood cell
LDH: Lactate dehydrogenase
NLR: Neutrophillymphocyte ratio
IRB: Institutional review board
CTCAE: Common terminology criteria adverse events
BMI: Body mass index
HR: Hazard ratio
CI: Confidence interval
ASCO: American Society of Clinical Oncology
AMC: Absolute monocyte count
IL-6: Interleukin-6
NSCLC: Non-small cell lung cancer.
